# Influence of Obesity and Unemployment on Fertility Rates: A Multinational Analysis of 30 Countries from 1976 to 2014

**DOI:** 10.3390/jcm11051152

**Published:** 2022-02-22

**Authors:** Deirdre Maria König-Castillo, Johannes Ott, Daniel König, Marlene Hager, Maike Katja Kahr, Georg Dorffner

**Affiliations:** 1 Department of Urology, Medical University of Vienna, 1090 Vienna, Austria; deirdre.koenig-castillo@meduniwien.ac.at; 2 Clinical Division of Gynecologic Endocrinology and Reproductive Medicine, Department of Obstetrics and Gynecology, Medical University of Vienna, 1090 Vienna, Austria; marlene.hager@meduniwien.ac.at; 3 Clinical Division of Social Psychiatry, Department of Psychiatry and Psychotherapy, Medical University of Vienna, 1090 Vienna, Austria; daniel.koenig-castillo@meduniwien.ac.at; 4 Department of Obstetrics, University Hospital of Zurich, CH-8091 Zurich, Switzerland; maike.kahr@gmail.com; 5 Institute for Artificial Intelligence, Medical University of Vienna, 1090 Vienna, Austria; georg.dorffner@meduniwien.ac.at

**Keywords:** fertility, overweight, unemployment

## Abstract

Background: The rationale of a postulated decrease in fertility rate development is still being debated. Among the multiple influencing factors, socioeconomic variables and their complex influence are of particular interest. Methods: Data on socioeconomic and health variables from 1976–2014 of 30 countries within the OECD region were analysed for their respective influence on fertility rates by using mixed-effect regression models. Results: A significant negative influence of the increase in unemployment rate on the following year’s changes in fertility rate in Western (−0.00256; *p* < 0.001) as well as Eastern European (−0.0034; *p* < 0.001) countries was revealed. The effect of being overweight was significant for Western European (−0.00256; *p* < 0.001) countries only. When analysing the whole OECD region, an increase in unemployment retained its significant negative influence on the fertility rate (−0.0028; *p* < 0.001), while being overweight did not. Interestingly, divergent influences of time were revealed and fertility rates increased with time in Eastern Europe while they decreased in Western Europe. Conclusion: Importantly, a significant negative influence of increase in unemployment on the fertility rate was revealed—irrespective of the region and time analysed. Furthermore, an adverse effect of being overweight on the fertility rate in Western European countries was revealed. Interestingly, time was associated with a decreasing fertility rate in Western but not in Eastern Europe.

## 1. Introduction

Disagreement on the rationale of the often-postulated declining fertility rates (defined as the number of children born per woman aged 15–49 years of age per year per region) of the recent decades in literature persists [1,2,3,4,5]. Notably, the World Health Organization (WHO) reports fluctuations in the fertility rates in Organization for Economic Co-operation and Development (OECD) countries for the previous four decades [6]. While a strong reduction in fertility rates for most countries is observable following the first introduction of the birth control pill in 1960 [7], later changes are less pronounced [6]. Conclusive results on a possible causal relationship with the various influencing factors are not established, and conflicting hypotheses have been postulated. One group of factors often said to influence the fertility rate within a society is economic conditions [1,5,8].

Previous literature on this topic revealed inconsistent and often conflicting results. While some studies reported a significant positive influence of being employed on fertility [1,2,5,9], other studies reported the opposite [3,4]. Örsal and Goldstein even suggested the influence of employment status on fertility rates to have changed its direction of influence in the 1970s: While an anticyclical relationship between fertility rates and employment was reported up until 1970 (i.e., increases in the unemployment rate were positively associated with increasing fertility rates), a pro-cyclical relationship was found for the data thereafter [8]. Moreover, recently, Puig-Barrachina et al. reported conflicting results on the effect of changes in the unemployment rate in Spain: while increases in the unemployment rate had a negative impact on fertility rates in some regions, this effect failed to be relevant in others. Furthermore, equally for other variables measuring the financial prosperity of affected countries (e.g., gross domestic product (GDP)), there is a lack of agreement about the influence on fertility [10]. While some studies reported a positive effect of increasing GDP on fertility rates [2], others reported a negative effect on fertility [11,12].

The effect of financial well-being is further complicated by its reported inverted “U-shape” association with body mass index (BMI)—with the highest rates of obesity being reported in women of average financial well-being. These rates gradually decrease towards the very low as well as very high range of financial well-being [13]. Obesity is hypothesised to be an important factor in women’s fertility. Notably, prevalence rates of obesity in U.S. women are reported to be increasing, with 23% of women of reproductive age being obese [14,15]. Literature suggests obesity to be a risk factor associated with a longer time until pregnancy [16,17,18]. Moreover, with a body mass index (BMI) over 29 kg/m^2^, the chance for a spontaneous pregnancy declined linearly [19]. In addition, also being overweight (BMI 25–29 kg/m^2^) is known to be a risk factor for lower fertility [16]. Thus, even with assisted reproductive technology (ART), obese women have a poorer chance of getting pregnant. Moreover, for obese women, poorer pregnancy outcomes are reported, as they suffer from miscarriage, stillbirth and preeclampsia more often [17,18].

As a lack of consensus on the rationale of declining fertility rates exists and complex interactions of health and financial well-being are postulated, our study aimed to investigate these interactions in a large, multinational cohort. To ensure comparability of data quality as well as health care systems, only data on countries partaking in the Organization for Economic Co-Operation and Development (OECD) were used. Data on socioeconomic variables as well as on health of the population for the last four decades were included. To the best of our knowledge, this is the first multicountry study investigating the influence of wealth and obesity on fertility rates.

## 2. Materials and Methods

### 2.1. Data

Data were retrieved from the “Organization for Economic Co-operation and Development Statistics Database” (OECD Stats Database) as well as the “World Health Organization—Health for All Database” (WHO-HFA) for the period of 1976–2014. The following data were collected for each country: the fertility rate (defined as the number of children born per woman aged 15–49 years of age); percentage of the population diagnosed as being overweight or obese (defined by a BMI of ≥25 kg/m^2^); socioeconomic data that included the unemployment rate (defined as the number of persons currently unemployed per 1000 of the population). Only those countries for which a complete dataset of the examined variables was available for the analysed period were included (allocation per UN definition [20]):

(i) Western European OECD Countries (taken from the ones listed in [8]): Austria, Belgium, Denmark, Finland, France, Germany, Greece, Ireland, Italy, Luxemburg, Netherlands, Norway, Portugal, Spain, Sweden, Switzerland, UK.
(ii) Eastern European OECD Countries: Czech Republic, Estonia, Hungary, Latvia, Poland, Slovak Republic, Slovenia.
(iii) Excluded (neither of the above groups or no data available on obesity): Australia, Canada, Chile, Iceland, Israel, Japan, Korea, Lithuania, Mexico, New Zealand, Turkey, United States.

### 2.2. Statistical Analysis

We roughly followed Örsal and Goldstein [8] and estimated a mixed-effect model for the logarithm of the fertility rate after differentiating all time-series to eliminate trends.

$$\Delta \ln TFR_{i,t}=\alpha +\mu_{i}+\nu_{i}g\left(t\right)+\beta_{1}\Delta x_{i,t-1}+\beta_{2}\Delta y_{i,t-1}+\beta_{3}\Delta z_{i,t-1}$$

where *TFR_i,t_* is the total fertility rate in country *I* at year *t*, and *x*, *y* and *z* are the unemployment rate, overweight and obesity rates, respectively. *µ_i_* and *ν_i_* are the random effects in country *i*. Δ is the differencing operator, such that

$$\Delta x_{i,t}=x_{i,t}-x_{i,t-1}$$

*G*(*t*) was set to *t*-1960, with *t* representing the year. As opposed to [8], we did not model interactions between this time variable and the economic indicators but added it as an additional predictor in the model. Models were calculated with all three independent variables or only a subset (by setting some of the *βi* to 0.)

For comparison purposes, we also took data from the same 22 OECD countries considering the period from 1976–2014. In addition, we chose the data from 8 Eastern European OECD countries and recalculated the models for those and the totality of 30 countries. All calculations were performed with the SAS University Edition software (2021), using the mixed model, and the level of significance was set at <0.05.

## 3. Results

### 3.1. Descriptive Analysis

**Figure 1:**
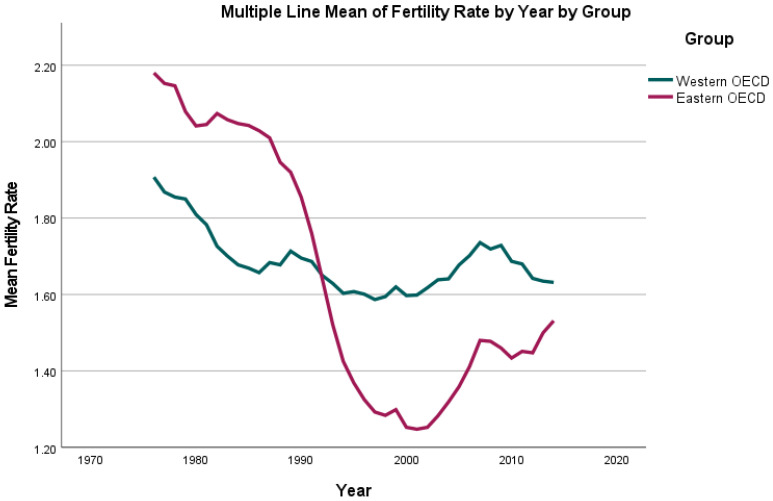
Mean fertility rate (children per woman) per country group (Eastern/Western Organization for Economic Co-operation and Development (OECD) countries) per year from 1976 until 2014.

There were pronounced differences in fertility rates across the 30 OECD countries (see Figure 1) included in the analysis: while the highest fertility rate was reported as 3.31 children per woman aged 15–49 years old per year for Ireland in 1976, the lowest was 1.1 for Latvia in 1998. For the included time series from 1976 until 2014, an ongoing reduction of fertility rate was observed for all countries with a mean of 2.03 children per woman aged 15–49 years old per year in 1976 and 1.60 children in 2014.

**Figure 2:**
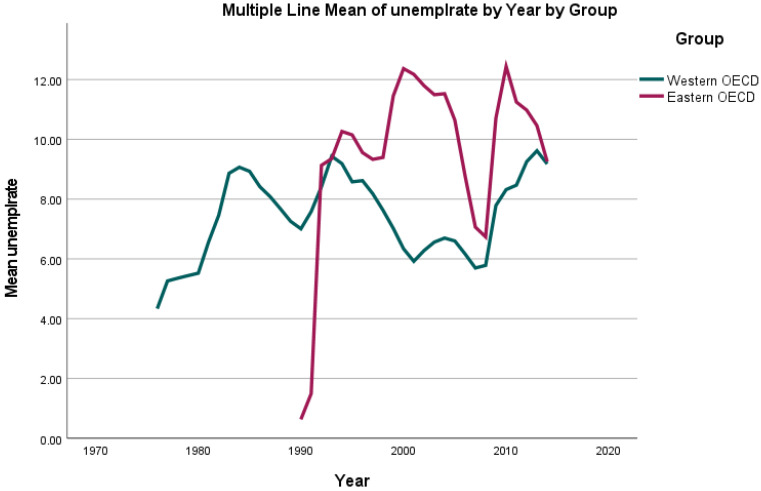
Mean unemployment rate (number of individuals of the population of working age registered as being unemployed) per country group (Eastern/Western OECD countries) per year from 1976 until 2014.

**Figure 3:**
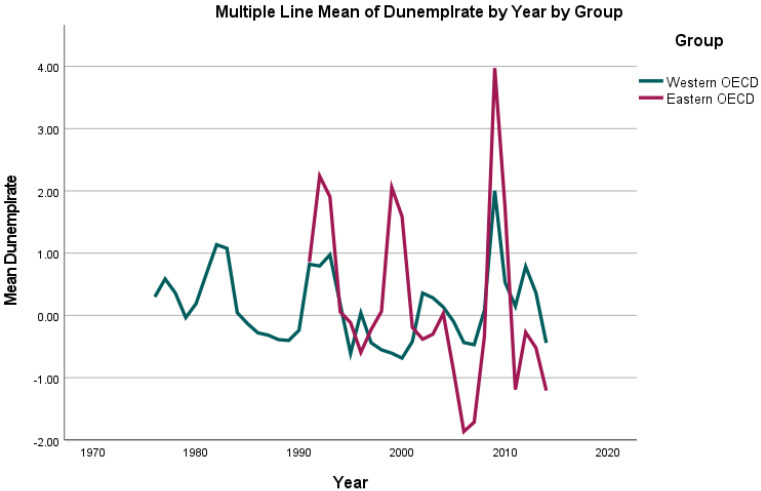
Mean change in unemployment rate (number of individuals of the population of working age registered as being unemployed) when compared to the prior year (“dUnemplrate”) per country group (Eastern/Western OECD countries) per year from 1976 until 2014.

For the included variables “overweight” (percentage of the population with a BMI of ≥25 kg/m^2^ aged 18 years and over) as well as “unemployment”, diverging trends were observed in our dataset: While the highest rates of unemployment (number of individuals of the population of working age registered as being unemployed per 1000 population) were reported as 14,827,000 in 2010 for the UK, the lowest (and most stable) rate was 2000 reported for Luxembourg in 1988–1992. For the above-mentioned period, the incidence of unemployment fluctuated (See Figure 2 and Figure 3) with a mean of 2,283,000 in 1976 and a mean of 1,247,000 in 2014.

**Figure 4:**
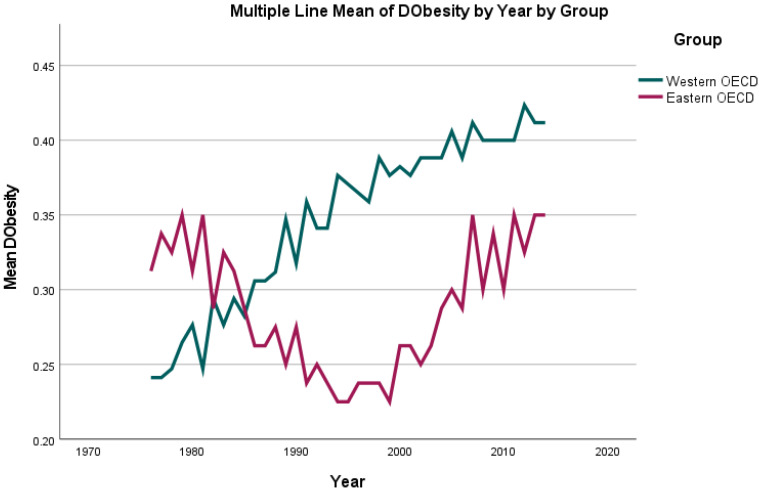
Mean change in percentage of the population diagnosed with obesity (BMI ≥ 30) when compared to the prior year (“dObesity”) per country group (Eastern/Western OECD countries) per year from 1976 until 2014.

**Figure 5:**
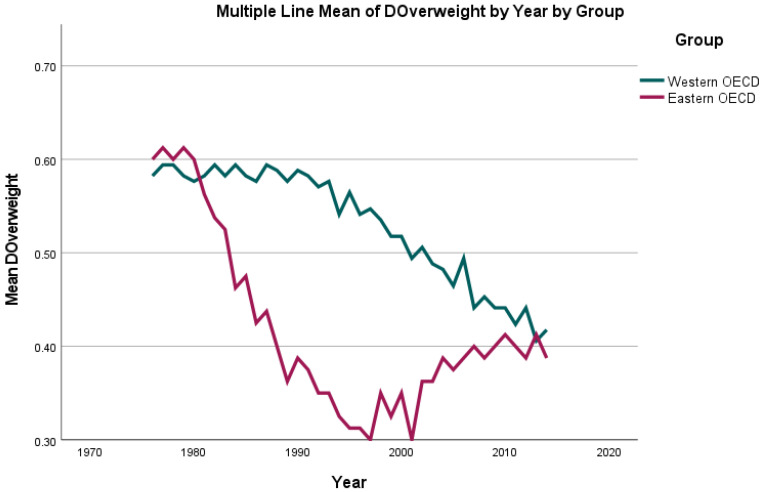
Mean change in percentage of the population diagnosed with being overweight (BMI ≥ 25) when compared to the prior year (“dOverweight”) per country group (Eastern/Western OECD countries) per year from 1976 until 2014.

While the highest percentage of the population diagnosed as suffering from overweight was reported as 62.7% for the UK in 2014, the lowest was reported as 31.1% for Switzerland in 1976. For the analysed period, an increasing percentage of the population diagnosed as being overweight was observed for all countries (see Figure 4 and Figure 5), with a mean of 38.91% in 1976 and 57.06% in 2014.

(Details are provided in Supplementary Tables).

### 3.2. Mixed Effects Regression Models

The statistical models, which included the variables overweight, obesity and unemployment rate after differentiation (termed “dOverweight”, “dObesity” and “dUnemplrate”), as well as year, revealed significant influences of these parameters on changes in fertility rates (“dFertility”) in the respective countries. The analyses were performed for Western and Eastern countries separately as well as all OECD countries combined. Details are provided in Table 1.

#### 3.2.1. Western OECD Countries

When the effect of the change in the percentage of population being overweight was analysed, a significant negative influence on the change in fertility rate of the following year was observed in the Western OECD countries (−0.0088; *p* < 0.01). Thus, an increase of one percent in the percentage of the population diagnosed with being overweight resulted in a decrease of the natural logarithm of the fertility rate by 0.0088 (e.g., a fertility rate of 1.7 children per woman would be decreasing to 1.6).

Furthermore, a significant negative influence for the variable “dUnemplrate” on the change in fertility rate of the affected countries was revealed in our analysis (−0.00256; *p* < 0.001). Thus, an increase of one percent in the percentage of the population being unemployed resulted in a decrease of fertility rate in the following year of 0.25%.

However, analysing the effect of time as a fixed effect on the fertility rate, our model failed to reveal any significant influence.

#### 3.2.2. Eastern OECD Countries

Contrary to our analysis of Western OECD countries, in our analysis of data from Eastern OECD countries, we failed to reveal a significant influence of the effect of change in rate of overweight of the affected population on the change in fertility rate of the following year (0.0242; *p* = ns).

However, when the effect of the change in the unemployment rate of the affected population on the change of fertility rate of the following year was analysed, a significant negative influence was also revealed for the Eastern OECD countries (−0.0034; *p* < 0.001).

Again, differing from the group of Western OECD countries, the variable time was revealed to exhibit a significant influence on the fertility rate of the affected population (0.000871; *p* < 0.05). Thus, for Eastern OECD countries, with ongoing time, fertility rate increased per year by 0.08%.

#### 3.2.3. Model including All Countries

The model, including data on all included OECD countries available (Western and Eastern countries combined) and analysing the possible influence of the variables overweight, unemployment and time, revealed a significant influence of “dUnemplrate” on the change in fertility rate on the respective countries included (−0.0028; *p* < 0.001). In addition, the variable time was revealed to exhibit a significant influence on the change in fertility rate of the affected population (0.00040; *p* < 0.01). Thus, with ongoing time, the fertility rate increased per year by 0.04%. However, the variable “dOverweight” failed to retain the previously described negative significant association the model had revealed for Western OECD countries.

## 4. Discussion

In our analysis of data on 30 countries for the period from 1976–2014 available at OECD Stats Database and WHO-HFA, an ongoing reduction in fertility rates across all included OECD countries was observed. Importantly, our analyses revealed a significant negative influence of the increase in unemployment rate on the following year’s fertility rate of the corresponding population in Western as well as Eastern European countries. However, from 1976 onwards, the effect of the percentage of the population diagnosed with being overweight was significant for Western European OECD countries only.

In our analysis, an increase of one percent in the unemployment rate corresponded to a decrease in the following year’s fertility rate by 0.28% (*p* < 0.001). Similar effects had been reported in the analyses of data in Europe and Latin America by Adsera et al. and Lee [2,5], where increases in unemployment rate corresponded to decreases in fertility rate. Other analyses, however, failed to reveal a similar effect of unemployment rate on fertility rate or reported contradictory findings [3,4]—e.g., Tejada et al. in Brazil, where being unemployed was reported to be associated with a higher number of children.

Interestingly, the seminal study by Örsal and Goldstein, which our work was based on, reported inconclusive results pre- and post-1975: While prior to 1975, the unemployment rates and the fertility rates were counter-cyclical (high unemployment rates were associated with high fertility rates), following 1975, the fertility rate and the unemployment rate were pro-cyclical (high unemployment rates were associated with low fertility rates). However, older data may be of limited quality or validity. Furthermore, the correlation changing its vector of association may be partly caused by the increasing availability of oral hormonal contraceptives [7] and increases in the availability of legal abortion [21]. These changes, in turn, may lead to limited comparability of these results and more recent data.

Analysis of our data supports the previously postulated hypothesis that couples may tend to decide against having children in more stressful and economically uncertain times and postpone the time of childbearing to times with better economic conditions [8]. However, the negative effect of a population’s unemployment rate is not limited to the short-term only: Currie and Schwandt have shown that women who are unemployed in their early twenties are more likely to remain childless or have fewer children [1].

However, our findings corroborate the fertility rate of a population to be dependent on a multitude of factors. Therefore, it is important to clarify that changes in fertility rates are not merely a reaction to unemployment. Furthermore, (fear of) unemployment and economic uncertainty cause stress [22], which is known to decrease fertility in women [23] by activating the hypothalamic-pituitary-adrenal (HPA) axis and thus cause increases in the glucocorticoid levels. Glucocorticoids have an inhibiting effect on the HPO axis [24]. Following this, stress causes menstrual irregularity [24] and decreases sexual desire in women [25]. Furthermore, stress causes erectile dysfunction in men [26]. Additionally, higher income is associated with a healthier lifestyle (measured in eating habits, physical activity and substance abuse) and illness-free life (measured as well in self-rated health as in the absence of chronic disease) [27,28].

In contrast to previous studies, both countries from Western as well as from Eastern Europe were included in the present analysis. Interestingly, the significant, negative effect of an increase in unemployment rate on the fertility rate of the corresponding population was more pronounced in Eastern countries (Eastern: −0.0034; *p* < 0.001, compared to Western: −0.0026; *p* < 0.001). We hypothesise couples in Eastern countries to be under more economic pressure, in part due to fewer funds available for welfare as well as the health care system. Thus, people in Eastern Europe may feel more insecure. To finance a family, it could be necessary to have both parents employed. In contrast, in Western Europe, a more robust health care system and a denser system of governmental social support may exist [29]. Thus, in Western countries, one working parent may be sufficient. Furthermore, social benefits might be better in Western countries. Hence, hypothetically, in Eastern Europe, unemployment and the threat of being unemployed might cause a higher stress level.

Interestingly, our data show a marked decline in fertility rate around the time of the collapse of Soviet Union: while prior to 1989 the fertility rate in Eastern Europe was higher than in Western Europe, a steep decline can be observed thereafter. Corroborating our findings, previous literature has described fertility rates to have declined in most of Eastern Europe following the disbandment of the former Soviet Union in 1989 [30]. Similarly, for Eastern and Western Europe, a stark reduction in fertility rate following the global economic crisis of 2008 can be observed in our data. These reductions in fertility rate may be hypothesised to be, in part, due to increased socioeconomic stress following a rise in unemployment rate or political upheaval.

To the best of our knowledge, this is the first study that included both health and socioeconomic variables. Considering the influence on fertility rate may be manifold and different influencing factors are indispensable. In our analysis of Western OECD countries, an increase in overweight of one percent corresponded to a decrease of 0.88% of fertility rate. The negative influence of the prevalence of overweight on fertility rate in our analysis was striking. Importantly, suffering from being overweight or obese is known to represent a risk for a decrease in fertility in females [16,19]. Considering that overweight and obesity are widespread phenomena with increasing prevalence [15], analysing the effect of overweight and obesity on fertility rate of a large population is of high interest.

In contrast to our data on Western OECD countries, overweight was not linked to the fertility rate in Eastern countries. Interestingly, the relationship between overweight and wealth is inconsistent: in wealthier and more developed countries, people with little financial security are more often overweight and obese; this association changes in less wealthy and developed countries. In these countries, the effect is inverse and persons with higher financial security are more often overweight and obese [31,32]. These differences in the distribution of the prevalence of overweight depending on the wealth of the analysed countries may have been an important confounding factor in our analysis. Thus, it may explain the difference in the prevalence of obesity and overweight on the fertility rate revealed in our analysis for Europe.

Several study limitations should be addressed. For the present analysis, we relied on administrative registry data. As these are limited to accuracy on a population level, no direct conclusions at the individual level may be drawn. Furthermore, the variable “overweight” was limited in resolution, as data were imputed as BMI > 25 kg/m^2^. Thus, the percentage of the population diagnosed with obesity (BMI > 30 kg/m^2^) was included in this variable, limiting the ability to analyse the possible distinction in fertility rates due to hypothesised changes in hormonal values. Furthermore, on an aggregate level, the fact that the availability of data for the two sexes separately was limited may, however, be of importance [33]. In addition, data were incomplete for unemployment rate for Eastern countries for the years 1976–1989. The difference in observations available for Eastern OECD countries when compared to the number of Western OECD countries may, thus, influence the estimated effect of the investigated variables. However, in contrast to previous studies, our analysis included a more diverse list of countries. We included not only high-income countries but also low-income countries. Furthermore, we included a broader spectrum of possible influencing factors by focusing on economics and health-related factors.

## 5. Conclusions

In conclusion, an ongoing reduction in fertility rates across all included OECD countries was observed. In all countries analysed as well as in the sub-analysis of Western OECD countries, a significant negative influence of the unemployment rate on the following year’s fertility rate was observed. From 1976 onwards, the effect of the percentage of the population diagnosed with being overweight was significant for Western European OECD countries only. Our findings underline the manifold influencing factors on fertility rates. The relationships are large-scale, and more multifactorial and multinational studies are required.

## Figures and Tables

**Table 1:** Mixed Effects Regression Model investigating the association of the variables “dUnemplrate”; “dOverweight” and “Year” with the change of fertility rate (“dFertility”) of the corresponding population for (1) Western European Countries, (2) Eastern European Countries and (3) All countries included in the analysis.

Variables	Western European Countries	Eastern European Countries	All included Countries
dUnemplrate	−0.0026 ***	−0.0034 ***	−0.0028 ***
dOverweight	−0.0088 **	0.0242	0.0006
Year	0.00015	0.00087 *	0.00040 **

Level of significance: * *p* < 0.05; ** *p* < 0.01; *** *p* < 0.001.

## Data Availability

All data is publicly available at https://stats.oecd.org and https://www.euro.who.int/en/data-and-evidence/databases/european-health-for-all-family-of-databases-hfa-db, accessed on 16 February 2022.

## Supplementary Materials

The following supporting information can be downloaded at: https://www.mdpi.com/article/10.3390/jcm11051152/s1, Table S1: Fertility Supplement, Figure S1: Fertility rate (children per women) per country per year from 1976 until 2014, Figure S2. Overweight rate per country per year from 1976 until 2014, Figure S3: Obesity rate per country per year from 1976 until 2014, Figure S4: Unemployment rate (number of individuals of the population of working age registered as being unemployed) per country per year from 1976 until 2014.
