# Supplementary material for: Influence of Obesity and Unemployment on Fertility Rates: A Multinational Analysis of 30 Countries from 1976 to 2014
Source: J Clin Med. 2022 Feb 22;11(5):1152. doi: 10.3390/jcm11051152 (PMC8911065; doi:10.3390/jcm11051152)
Supplement: Supplementary file 1 [file jcm-11-01152-s001.zip › jcm-1506717-supplementary.pdf]

Table S1. Case Processing Summary

		Cases					
		Valid		Missing		Total	
	Country	N	Percent	N	Percent	N	Percent
Fertility	Austria	21	53,8%	18	46,2%	39	100,0%
	Belgium	32	82,1%	7	17,9%	39	100,0%
	Czech Re	22	56,4%	17	43,6%	39	100,0%
	Denmark	32	82,1%	7	17,9%	39	100,0%
	Estonia	25	64,1%	14	35,9%	39	100,0%
	Finland	39	100,0%	0	0,0%	39	100,0%
	France	39	100,0%	0	0,0%	39	100,0%
	Germany	39	100,0%	0	0,0%	39	100,0%
	Greece	32	82,1%	7	17,9%	39	100,0%
	Hungary	23	59,0%	16	41,0%	39	100,0%
	Ireland	35	89,7%	4	10,3%	39	100,0%
	Italy	39	100,0%	0	0,0%	39	100,0%
	Latvia	15	38,5%	24	61,5%	39	100,0%
	Luxembou	32	82,1%	7	17,9%	39	100,0%
	Netherla	39	100,0%	0	0,0%	39	100,0%
	Norway	39	100,0%	0	0,0%	39	100,0%
	Poland	23	59,0%	16	41,0%	39	100,0%
	Portugal	39	100,0%	0	0,0%	39	100,0%
	Slovak R	21	53,8%	18	46,2%	39	100,0%
	Slovenia	15	38,5%	24	61,5%	39	100,0%
	Spain	39	100,0%	0	0,0%	39	100,0%
	Sweden	39	100,0%	0	0,0%	39	100,0%
	Switzerl	24	61,5%	15	38,5%	39	100,0%
	United K	31	79,5%	8	20,5%	39	100,0%
Overweight	Austria	21	53,8%	18	46,2%	39	100,0%
	Belgium	32	82,1%	7	17,9%	39	100,0%
	Czech Re	22	56,4%	17	43,6%	39	100,0%
	Denmark	32	82,1%	7	17,9%	39	100,0%
	Estonia	25	64,1%	14	35,9%	39	100,0%
	Finland	39	100,0%	0	0,0%	39	100,0%
	France	39	100,0%	0	0,0%	39	100,0%
	Germany	39	100,0%	0	0,0%	39	100,0%
	Greece	32	82,1%	7	17,9%	39	100,0%
	Hungary	23	59,0%	16	41,0%	39	100,0%
	Ireland	35	89,7%	4	10,3%	39	100,0%
	Italy	39	100,0%	0	0,0%	39	100,0%
	Latvia	15	38,5%	24	61,5%	39	100,0%

Case Processing Summary

	Country	Cases					
		Valid		Missing		Total	
		N	Percent	N	Percent	N	Percent
	Luxembou	32	82,1%	7	17,9%	39	100,0%
	Netherla	39	100,0%	0	0,0%	39	100,0%
	Norway	39	100,0%	0	0,0%	39	100,0%
	Poland	23	59,0%	16	41,0%	39	100,0%
	Portugal	39	100,0%	0	0,0%	39	100,0%
	Slovak R	21	53,8%	18	46,2%	39	100,0%
	Slovenia	15	38,5%	24	61,5%	39	100,0%
	Spain	39	100,0%	0	0,0%	39	100,0%
	Sweden	39	100,0%	0	0,0%	39	100,0%
	Switzerl	24	61,5%	15	38,5%	39	100,0%
	United K	31	79,5%	8	20,5%	39	100,0%
Obesity	Austria	21	53,8%	18	46,2%	39	100,0%
	Belgium	32	82,1%	7	17,9%	39	100,0%
	Czech Re	22	56,4%	17	43,6%	39	100,0%
	Denmark	32	82,1%	7	17,9%	39	100,0%
	Estonia	25	64,1%	14	35,9%	39	100,0%
	Finland	39	100,0%	0	0,0%	39	100,0%
	France	39	100,0%	0	0,0%	39	100,0%
	Germany	39	100,0%	0	0,0%	39	100,0%
	Greece	32	82,1%	7	17,9%	39	100,0%
	Hungary	23	59,0%	16	41,0%	39	100,0%
	Ireland	35	89,7%	4	10,3%	39	100,0%
	Italy	39	100,0%	0	0,0%	39	100,0%
	Latvia	15	38,5%	24	61,5%	39	100,0%
	Luxembou	32	82,1%	7	17,9%	39	100,0%
	Netherla	39	100,0%	0	0,0%	39	100,0%
	Norway	39	100,0%	0	0,0%	39	100,0%
	Poland	23	59,0%	16	41,0%	39	100,0%
	Portugal	39	100,0%	0	0,0%	39	100,0%
	Slovak R	21	53,8%	18	46,2%	39	100,0%
	Slovenia	15	38,5%	24	61,5%	39	100,0%
	Spain	39	100,0%	0	0,0%	39	100,0%
	Sweden	39	100,0%	0	0,0%	39	100,0%
	Switzerl	24	61,5%	15	38,5%	39	100,0%
	United K	31	79,5%	8	20,5%	39	100,0%
unemplrate	Austria	21	53,8%	18	46,2%	39	100,0%
	Belgium	32	82,1%	7	17,9%	39	100,0%

Case Processing Summary

Country	Cases					
	Valid		Missing		Total	
	N	Percent	N	Percent	N	Percent
Czech Re	22	56,4%	17	43,6%	39	100,0%
Denmark	32	82,1%	7	17,9%	39	100,0%
Estonia	25	64,1%	14	35,9%	39	100,0%
Finland	39	100,0%	0	0,0%	39	100,0%
France	39	100,0%	0	0,0%	39	100,0%
Germany	39	100,0%	0	0,0%	39	100,0%
Greece	32	82,1%	7	17,9%	39	100,0%
Hungary	23	59,0%	16	41,0%	39	100,0%
Ireland	35	89,7%	4	10,3%	39	100,0%
Italy	39	100,0%	0	0,0%	39	100,0%
Latvia	15	38,5%	24	61,5%	39	100,0%
Luxembou	32	82,1%	7	17,9%	39	100,0%
Netherla	39	100,0%	0	0,0%	39	100,0%
Norway	39	100,0%	0	0,0%	39	100,0%
Poland	23	59,0%	16	41,0%	39	100,0%
Portugal	39	100,0%	0	0,0%	39	100,0%
Slovak R	21	53,8%	18	46,2%	39	100,0%
Slovenia	15	38,5%	24	61,5%	39	100,0%
Spain	39	100,0%	0	0,0%	39	100,0%
Sweden	39	100,0%	0	0,0%	39	100,0%
Switzerl	24	61,5%	15	38,5%	39	100,0%
United K	31	79,5%	8	20,5%	39	100,0%

Descriptives[a,b,c,d]

	Country			Statistic	Std. Error
Fertility	Austria	Mean		1,4086	,00865
		95% Confidence Interval for Mean	Lower Bound	1,3905	
			Upper Bound	1,4266	
		5% Trimmed Mean		1,4085	
		Median		1,4100	
		Variance		,002	
		Std. Deviation		,03966	
		Minimum		1,33	
		Maximum		1,49	
		Range		,16	
		Interquartile Range		,06	
		Skewness		-,149	,501
		Kurtosis		-,065	,972
	Belgium	Mean		1,6584	,01829
		95% Confidence Interval for Mean	Lower Bound	1,6211	
			Upper Bound	1,6957	
		5% Trimmed Mean		1,6553	
		Median		1,6400	
		Variance		,011	
		Std. Deviation		,10349	
		Minimum		1,51	
		Maximum		1,86	
		Range		,35	
		Interquartile Range		,17	
		Skewness		,529	,414
		Kurtosis		-,999	,809
	Czech Re	Mean		1,3277	,03253
		95% Confidence Interval for Mean	Lower Bound	1,2601	
			Upper Bound	1,3954	
		5% Trimmed Mean		1,3254	
		Median		1,3050	
		Variance		,023	
		Std. Deviation		,15256	
		Minimum		1,13	
		Maximum		1,57	
		Range		,44	
		Interquartile Range		,30	
		Skewness		,069	,491

Descriptives[a,b,c,d]

Country				Statistic	Std. Error
		Kurtosis		-1,717	,953
	Denmark	Mean		1,7156	,02153
		95% Confidence Interval for Mean	Lower Bound	1,6717	
			Upper Bound	1,7595	
		5% Trimmed Mean		1,7228	
		Median		1,7500	
		Variance		,015	
		Std. Deviation		,12181	
		Minimum		1,40	
		Maximum		1,89	
		Range		,49	
		Interquartile Range		,13	
		Skewness		-1,135	,414
		Kurtosis		,846	,809
	Estonia	Mean		1,5072	,03129
		95% Confidence Interval for Mean	Lower Bound	1,4426	
			Upper Bound	1,5718	
		5% Trimmed Mean		1,5042	
		Median		1,5200	
		Variance		,024	
		Std. Deviation		,15643	
		Minimum		1,28	
		Maximum		1,80	
		Range		,52	
		Interquartile Range		,29	
		Skewness		,229	,464
		Kurtosis		-1,192	,902
	Finland	Mean		1,7449	,01242
		95% Confidence Interval for Mean	Lower Bound	1,7197	
			Upper Bound	1,7700	
		5% Trimmed Mean		1,7465	
		Median		1,7400	
		Variance		,006	
		Std. Deviation		,07756	
		Minimum		1,59	
		Maximum		1,87	
		Range		,28	
		Interquartile Range		,11	

Descriptives[a,b,c,d]

Country				Statistic	Std. Error
		Skewness		-,194	,378
		Kurtosis		-,911	,741
	France	Mean		1,8559	,01601
		95% Confidence Interval for Mean	Lower Bound	1,8235	
			Upper Bound	1,8883	
		5% Trimmed Mean		1,8582	
		Median		1,8600	
		Variance		,010	
		Std. Deviation		,09999	
		Minimum		1,66	
		Maximum		2,02	
		Range		,36	
		Interquartile Range		,17	
		Skewness		-,131	,378
		Kurtosis		-,964	,741
	Germany	Mean		1,3949	,01229
		95% Confidence Interval for Mean	Lower Bound	1,3700	
			Upper Bound	1,4197	
		5% Trimmed Mean		1,3948	
		Median		1,3800	
		Variance		,006	
		Std. Deviation		,07674	
		Minimum		1,24	
		Maximum		1,56	
		Range		,32	
		Interquartile Range		,11	
		Skewness		,203	,378
		Kurtosis		-,343	,741
	Greece	Mean		1,3819	,02384
		95% Confidence Interval for Mean	Lower Bound	1,3332	
			Upper Bound	1,4305	
		5% Trimmed Mean		1,3687	
		Median		1,3400	
		Variance		,018	
		Std. Deviation		,13487	
		Minimum		1,23	
		Maximum		1,82	
		Range		,59	

Descriptives[a,b,c,d]

Country				Statistic	Std. Error
		Interquartile Range		,18	
		Skewness		1,531	,414
		Kurtosis		2,635	,809
	Hungary	Mean		1,3726	,02439
		95% Confidence Interval for Mean	Lower Bound	1,3220	
			Upper Bound	1,4232	
		5% Trimmed Mean		1,3626	
		Median		1,3300	
		Variance		,014	
		Std. Deviation		,11698	
		Minimum		1,24	
		Maximum		1,69	
		Range		,45	
		Interquartile Range		,10	
		Skewness		1,642	,481
		Kurtosis		2,152	,935
	Ireland	Mean		2,1354	,06089
		95% Confidence Interval for Mean	Lower Bound	2,0117	
			Upper Bound	2,2592	
		5% Trimmed Mean		2,0902	
		Median		1,9900	
		Variance		,130	
		Std. Deviation		,36022	
		Minimum		1,85	
		Maximum		3,24	
		Range		1,39	
		Interquartile Range		,18	
		Skewness		2,145	,398
		Kurtosis		4,006	,778
	Italy	Mean		1,3992	,02839
		95% Confidence Interval for Mean	Lower Bound	1,3418	
			Upper Bound	1,4567	
		5% Trimmed Mean		1,3813	
		Median		1,3600	
		Variance		,031	
		Std. Deviation		,17728	
		Minimum		1,19	
		Maximum		1,97	

Descriptives[a,b,c,d]

Country				Statistic	Std. Error
		Range		,78	
		Interquartile Range		,15	
		Skewness		1,630	,378
		Kurtosis		2,702	,741
	Latvia	Mean		1,4347	,03695
		95% Confidence Interval for Mean	Lower Bound	1,3554	
			Upper Bound	1,5139	
		5% Trimmed Mean		1,4319	
		Median		1,4400	
		Variance		,020	
		Std. Deviation		,14312	
		Minimum		1,22	
		Maximum		1,70	
		Range		,48	
		Interquartile Range		,22	
		Skewness		,342	,580
		Kurtosis		-,744	1,121
	Luxembou	Mean		1,5975	,01837
		95% Confidence Interval for Mean	Lower Bound	1,5600	
			Upper Bound	1,6350	
		5% Trimmed Mean		1,5997	
		Median		1,6200	
		Variance		,011	
		Std. Deviation		,10389	
		Minimum		1,38	
		Maximum		1,78	
		Range		,40	
		Interquartile Range		,16	
		Skewness		-,491	,414
		Kurtosis		-,370	,809
	Netherla	Mean		1,6308	,01495
		95% Confidence Interval for Mean	Lower Bound	1,6005	
			Upper Bound	1,6610	
		5% Trimmed Mean		1,6300	
		Median		1,6100	
		Variance		,009	
		Std. Deviation		,09337	
		Minimum		1,47	

Descriptives[a,b,c,d]

Country				Statistic	Std. Error
		Maximum		1,80	
		Range		,33	
		Interquartile Range		,16	
		Skewness		,173	,378
		Kurtosis		-1,250	,741
	Norway	Mean		1,8174	,01384
		95% Confidence Interval for Mean	Lower Bound	1,7894	
			Upper Bound	1,8454	
		5% Trimmed Mean		1,8177	
		Median		1,8400	
		Variance		,007	
		Std. Deviation		,08641	
		Minimum		1,66	
		Maximum		1,98	
		Range		,32	
		Interquartile Range		,14	
		Skewness		-,112	,378
		Kurtosis		-,929	,741
	Poland	Mean		1,3757	,03070
		95% Confidence Interval for Mean	Lower Bound	1,3120	
			Upper Bound	1,4393	
		5% Trimmed Mean		1,3627	
		Median		1,3200	
		Variance		,022	
		Std. Deviation		,14724	
		Minimum		1,22	
		Maximum		1,77	
		Range		,55	
		Interquartile Range		,14	
		Skewness		1,488	,481
		Kurtosis		1,874	,935
	Portugal	Mean		1,5967	,05002
		95% Confidence Interval for Mean	Lower Bound	1,4954	
			Upper Bound	1,6979	
		5% Trimmed Mean		1,5739	
		Median		1,4800	
		Variance		,098	
		Std. Deviation		,31236	

Descriptives[a,b,c,d]

Country				Statistic	Std. Error
		Minimum		1,21	
		Maximum		2,48	
		Range		1,27	
		Interquartile Range		,26	
		Skewness		1,320	,378
		Kurtosis		,916	,741
	Slovak R	Mean		1,3338	,02090
		95% Confidence Interval for Mean	Lower Bound	1,2902	
			Upper Bound	1,3774	
		5% Trimmed Mean		1,3316	
		Median		1,3400	
		Variance		,009	
		Std. Deviation		,09578	
		Minimum		1,19	
		Maximum		1,52	
		Range		,33	
		Interquartile Range		,16	
		Skewness		,118	,501
		Kurtosis		-,929	,972
	Slovenia	Mean		1,4193	,04150
		95% Confidence Interval for Mean	Lower Bound	1,3303	
			Upper Bound	1,5083	
		5% Trimmed Mean		1,4226	
		Median		1,5300	
		Variance		,026	
		Std. Deviation		,16073	
		Minimum		1,20	
		Maximum		1,58	
		Range		,38	
		Interquartile Range		,32	
		Skewness		-,324	,580
		Kurtosis		-1,940	1,121
	Spain	Mean		1,4972	,06213
		95% Confidence Interval for Mean	Lower Bound	1,3714	
			Upper Bound	1,6230	
		5% Trimmed Mean		1,4540	
		Median		1,3400	
		Variance		,151	

Descriptives[a,b,c,d]

Country				Statistic	Std. Error
		Std. Deviation		,38800	
		Minimum		1,16	
		Maximum		2,67	
		Range		1,51	
		Interquartile Range		,29	
		Skewness		1,795	,378
		Kurtosis		2,465	,741
	Sweden	Mean		1,7836	,02829
		95% Confidence Interval for Mean	Lower Bound	1,7263	
			Upper Bound	1,8409	
		5% Trimmed Mean		1,7798	
		Median		1,7700	
		Variance		,031	
		Std. Deviation		,17669	
		Minimum		1,50	
		Maximum		2,14	
		Range		,64	
		Interquartile Range		,28	
		Skewness		,226	,378
		Kurtosis		-,905	,741
	Switzerl	Mean		1,4817	,01078
		95% Confidence Interval for Mean	Lower Bound	1,4594	
			Upper Bound	1,5040	
		5% Trimmed Mean		1,4821	
		Median		1,4850	
		Variance		,003	
		Std. Deviation		,05281	
		Minimum		1,38	
		Maximum		1,58	
		Range		,20	
		Interquartile Range		,08	
		Skewness		-,429	,472
		Kurtosis		-,391	,918
	United K	Mean		1,7826	,01480
		95% Confidence Interval for Mean	Lower Bound	1,7523	
			Upper Bound	1,8128	
		5% Trimmed Mean		1,7834	
		Median		1,7900	

Descriptives[a,b,c,d]

	Country			Statistic	Std. Error
		Variance		,007	
		Std. Deviation		,08242	
		Minimum		1,63	
		Maximum		1,92	
		Range		,29	
		Interquartile Range		,11	
		Skewness		-,095	,421
		Kurtosis		-,532	,821
Overweight	Austria	Mean		49,1571	,60125
		95% Confidence Interval for Mean	Lower Bound	47,9030	
			Upper Bound	50,4113	
		5% Trimmed Mean		49,1799	
		Median		49,3000	
		Variance		7,592	
		Std. Deviation		2,75528	
		Minimum		44,50	
		Maximum		53,40	
		Range		8,90	
		Interquartile Range		4,85	
		Skewness		-,123	,501
		Kurtosis		-1,178	,972
	Belgium	Mean		53,5844	,63410
		95% Confidence Interval for Mean	Lower Bound	52,2911	
			Upper Bound	54,8776	
		5% Trimmed Mean		53,6417	
		Median		53,9000	
		Variance		12,867	
		Std. Deviation		3,58699	
		Minimum		47,20	
		Maximum		58,90	
		Range		11,70	
		Interquartile Range		6,33	
		Skewness		-,223	,414
		Kurtosis		-1,184	,809
	Czech Re	Mean		58,0455	,45106
		95% Confidence Interval for Mean	Lower Bound	57,1074	
			Upper Bound	58,9835	
		5% Trimmed Mean		58,0232	

Descriptives[a,b,c,d]

Country				Statistic	Std. Error
		Median		57,9000	
		Variance		4,476	
		Std. Deviation		2,11564	
		Minimum		54,90	
		Maximum		61,60	
		Range		6,70	
		Interquartile Range		3,77	
		Skewness		,157	,491
		Kurtosis		-1,228	,953
	Denmark	Mean		48,3438	,73772
		95% Confidence Interval for Mean	Lower Bound	46,8392	
			Upper Bound	49,8483	
		5% Trimmed Mean		48,4021	
		Median		48,7000	
		Variance		17,415	
		Std. Deviation		4,17318	
		Minimum		40,90	
		Maximum		54,70	
		Range		13,80	
		Interquartile Range		7,27	
		Skewness		-,196	,414
		Kurtosis		-1,160	,809
	Estonia	Mean		51,5120	,40720
		95% Confidence Interval for Mean	Lower Bound	50,6716	
			Upper Bound	52,3524	
		5% Trimmed Mean		51,4811	
		Median		51,3000	
		Variance		4,145	
		Std. Deviation		2,03599	
		Minimum		48,50	
		Maximum		55,10	
		Range		6,60	
		Interquartile Range		3,60	
		Skewness		,228	,464
		Kurtosis		-1,169	,902
	Finland	Mean		48,1333	1,05657
		95% Confidence Interval for Mean	Lower Bound	45,9944	
			Upper Bound	50,2722	

Descriptives[a,b,c,d]

Country			Statistic	Std. Error
	5% Trimmed Mean		48,3095	
	Median		49,3000	
	Variance		43,537	
	Std. Deviation		6,59826	
	Minimum		35,70	
	Maximum		57,20	
	Range		21,50	
	Interquartile Range		11,60	
	Skewness		-,378	,378
	Kurtosis		-1,122	,741
France	Mean		49,5974	,88163
	95% Confidence Interval for Mean	Lower Bound	47,8127	
		Upper Bound	51,3822	
	5% Trimmed Mean		49,6056	
	Median		49,6000	
	Variance		30,313	
	Std. Deviation		5,50576	
	Minimum		40,40	
	Maximum		58,60	
	Range		18,20	
	Interquartile Range		9,80	
	Skewness		-,010	,378
	Kurtosis		-1,230	,741
Germany	Mean		48,0615	,84336
	95% Confidence Interval for Mean	Lower Bound	46,3543	
		Upper Bound	49,7688	
	5% Trimmed Mean		48,1352	
	Median		48,6000	
	Variance		27,739	
	Std. Deviation		5,26676	
	Minimum		38,70	
	Maximum		56,00	
	Range		17,30	
	Interquartile Range		9,30	
	Skewness		-,203	,378
	Kurtosis		-1,212	,741
Greece	Mean		54,0750	,83384

Descriptives[a,b,c,d]

Country				Statistic	Std. Error
		95% Confidence Interval for Mean	Lower Bound	52,3744	
			Upper Bound	55,7756	
		5% Trimmed Mean		54,1201	
		Median		54,3000	
		Variance		22,249	
		Std. Deviation		4,71689	
		Minimum		45,80	
		Maximum		61,50	
		Range		15,70	
		Interquartile Range		8,25	
		Skewness		-,130	,414
		Kurtosis		-1,161	,809
	Hungary	Mean		55,2652	,62290
		95% Confidence Interval for Mean	Lower Bound	53,9734	
			Upper Bound	56,5570	
		5% Trimmed Mean		55,2128	
		Median		54,9000	
		Variance		8,924	
		Std. Deviation		2,98734	
		Minimum		50,90	
		Maximum		60,60	
		Range		9,70	
		Interquartile Range		5,30	
		Skewness		,279	,481
		Kurtosis		-1,144	,935
	Ireland	Mean		48,5657	1,18011
		95% Confidence Interval for Mean	Lower Bound	46,1674	
			Upper Bound	50,9640	
		5% Trimmed Mean		48,6817	
		Median		48,8000	
		Variance		48,743	
		Std. Deviation		6,98165	
		Minimum		35,20	
		Maximum		59,50	
		Range		24,30	
		Interquartile Range		12,10	
		Skewness		-,155	,398
		Kurtosis		-1,083	,778

Descriptives[a,b,c,d]

Country				Statistic	Std. Error
Italy	Mean			49,0513	,85118
	95% Confidence Interval for Mean	Lower Bound		47,3282	
		Upper Bound		50,7744	
	5% Trimmed Mean			49,0765	
	Median			49,1000	
	Variance			28,256	
	Std. Deviation			5,31561	
	Minimum			40,00	
	Maximum			57,60	
	Range			17,60	
	Interquartile Range			9,30	
	Skewness			-,044	,378
	Kurtosis			-1,214	,741
Latvia	Mean			54,7867	,38777
	95% Confidence Interval for Mean	Lower Bound		53,9550	
		Upper Bound		55,6184	
	5% Trimmed Mean			54,7796	
	Median			54,8000	
	Variance			2,256	
	Std. Deviation			1,50184	
	Minimum			52,50	
	Maximum			57,20	
	Range			4,70	
	Interquartile Range			2,70	
	Skewness			,054	,580
	Kurtosis			-1,198	1,121
Luxembou	Mean			50,4438	,84151
	95% Confidence Interval for Mean	Lower Bound		48,7275	
		Upper Bound		52,1600	
	5% Trimmed Mean			50,4778	
	Median			50,6500	
	Variance			22,661	
	Std. Deviation			4,76032	
	Minimum			42,30	
	Maximum			57,90	
	Range			15,60	
	Interquartile Range			8,47	
	Skewness			-,089	,414

Descriptives[a,b,c,d]

Country				Statistic	Std. Error
		Kurtosis		-1,228	,809
	Netherla	Mean		45,5487	1,15527
		95% Confidence Interval for Mean	Lower Bound	43,2100	
			Upper Bound	47,8874	
		5% Trimmed Mean		45,5709	
		Median		45,6000	
		Variance		52,051	
		Std. Deviation		7,21464	
		Minimum		33,70	
		Maximum		56,90	
		Range		23,20	
		Interquartile Range		13,00	
		Skewness		-,030	,378
		Kurtosis		-1,284	,741
	Norway	Mean		47,5590	1,01523
		95% Confidence Interval for Mean	Lower Bound	45,5038	
			Upper Bound	49,6142	
		5% Trimmed Mean		47,6185	
		Median		47,7000	
		Variance		40,197	
		Std. Deviation		6,34009	
		Minimum		36,50	
		Maximum		57,40	
		Range		20,90	
		Interquartile Range		11,20	
		Skewness		-,101	,378
		Kurtosis		-1,230	,741
	Poland	Mean		52,6478	,59484
		95% Confidence Interval for Mean	Lower Bound	51,4142	
			Upper Bound	53,8814	
		5% Trimmed Mean		52,6309	
		Median		52,5000	
		Variance		8,138	
		Std. Deviation		2,85273	
		Minimum		48,20	
		Maximum		57,40	
		Range		9,20	
		Interquartile Range		5,10	

Descriptives[a,b,c,d]

Country				Statistic	Std. Error
		Skewness		,095	,481
		Kurtosis		-1,210	,935
	Portugal	Mean		44,2308	1,23168
		95% Confidence Interval for Mean	Lower Bound	41,7374	
			Upper Bound	46,7242	
		5% Trimmed Mean		44,2564	
		Median		44,3000	
		Variance		59,165	
		Std. Deviation		7,69187	
		Minimum		31,50	
		Maximum		56,50	
		Range		25,00	
		Interquartile Range		13,90	
		Skewness		-,042	,378
		Kurtosis		-1,273	,741
	Slovak R	Mean		51,3476	,52832
		95% Confidence Interval for Mean	Lower Bound	50,2456	
			Upper Bound	52,4497	
		5% Trimmed Mean		51,3418	
		Median		51,3000	
		Variance		5,862	
		Std. Deviation		2,42108	
		Minimum		47,50	
		Maximum		55,30	
		Range		7,80	
		Interquartile Range		4,30	
		Skewness		,044	,501
		Kurtosis		-1,209	,972
	Slovenia	Mean		52,0933	,52012
		95% Confidence Interval for Mean	Lower Bound	50,9778	
			Upper Bound	53,2089	
		5% Trimmed Mean		52,0926	
		Median		52,1000	
		Variance		4,058	
		Std. Deviation		2,01440	
		Minimum		49,00	
		Maximum		55,20	
		Range		6,20	

Descriptives[a,b,c,d]

Country				Statistic	Std. Error
		Interquartile Range		3,60	
		Skewness		,011	,580
		Kurtosis		-1,253	1,121
	Spain	Mean		52,3692	,91781
		95% Confidence Interval for Mean	Lower Bound	50,5112	
			Upper Bound	54,2272	
		5% Trimmed Mean		52,4855	
		Median		52,9000	
		Variance		32,853	
		Std. Deviation		5,73173	
		Minimum		41,60	
		Maximum		60,90	
		Range		19,30	
		Interquartile Range		9,80	
		Skewness		-,266	,378
		Kurtosis		-1,095	,741
	Sweden	Mean		47,0538	,85172
		95% Confidence Interval for Mean	Lower Bound	45,3296	
			Upper Bound	48,7781	
		5% Trimmed Mean		47,0738	
		Median		47,1000	
		Variance		28,291	
		Std. Deviation		5,31898	
		Minimum		38,10	
		Maximum		55,60	
		Range		17,50	
		Interquartile Range		9,50	
		Skewness		-,041	,378
		Kurtosis		-1,251	,741
	Switzerl	Mean		48,7792	,64712
		95% Confidence Interval for Mean	Lower Bound	47,4405	
			Upper Bound	50,1178	
		5% Trimmed Mean		48,8509	
		Median		49,0000	
		Variance		10,050	
		Std. Deviation		3,17024	
		Minimum		42,70	
		Maximum		53,50	

Descriptives[a,b,c,d]

	Country			Statistic	Std. Error
		Range		10,80	
		Interquartile Range		5,25	
		Skewness		-,299	,472
		Kurtosis		-,936	,918
	United K	Mean		54,1097	,96635
		95% Confidence Interval for Mean	Lower Bound	52,1361	
			Upper Bound	56,0832	
		5% Trimmed Mean		54,1219	
		Median		54,2000	
		Variance		28,949	
		Std. Deviation		5,38042	
		Minimum		45,30	
		Maximum		62,70	
		Range		17,40	
		Interquartile Range		9,60	
		Skewness		-,032	,421
		Kurtosis		-1,253	,821
Obesity	Austria	Mean		15,4571	,49201
		95% Confidence Interval for Mean	Lower Bound	14,4308	
			Upper Bound	16,4835	
		5% Trimmed Mean		15,4468	
		Median		15,4000	
		Variance		5,084	
		Std. Deviation		2,25468	
		Minimum		11,90	
		Maximum		19,20	
		Range		7,30	
		Interquartile Range		4,00	
		Skewness		,068	,501
		Kurtosis		-1,195	,972
	Belgium	Mean		16,6250	,49353
		95% Confidence Interval for Mean	Lower Bound	15,6184	
			Upper Bound	17,6316	
		5% Trimmed Mean		16,6042	
		Median		16,5500	
		Variance		7,794	
		Std. Deviation		2,79181	
		Minimum		12,30	

Descriptives[a,b,c,d]

Country				Statistic	Std. Error
		Maximum		21,40	
		Range		9,10	
		Interquartile Range		5,05	
		Skewness		,084	,414
		Kurtosis		-1,233	,809
	Czech Re	Mean		22,0455	,38572
		95% Confidence Interval for Mean	Lower Bound	21,2433	
			Upper Bound	22,8476	
		5% Trimmed Mean		22,0177	
		Median		21,8500	
		Variance		3,273	
		Std. Deviation		1,80916	
		Minimum		19,40	
		Maximum		25,20	
		Range		5,80	
		Interquartile Range		3,23	
		Skewness		,226	,491
		Kurtosis		-1,167	,953
	Denmark	Mean		13,7125	,52324
		95% Confidence Interval for Mean	Lower Bound	12,6453	
			Upper Bound	14,7797	
		5% Trimmed Mean		13,6826	
		Median		13,5500	
		Variance		8,761	
		Std. Deviation		2,95992	
		Minimum		9,10	
		Maximum		18,90	
		Range		9,80	
		Interquartile Range		5,22	
		Skewness		,131	,414
		Kurtosis		-1,182	,809
	Estonia	Mean		17,9480	,30156
		95% Confidence Interval for Mean	Lower Bound	17,3256	
			Upper Bound	18,5704	
		5% Trimmed Mean		17,9211	
		Median		17,8000	
		Variance		2,273	
		Std. Deviation		1,50779	

Descriptives[a,b,c,d]

Country				Statistic	Std. Error
		Minimum		15,70	
		Maximum		20,70	
		Range		5,00	
		Interquartile Range		2,65	
		Skewness		,270	,464
		Kurtosis		-1,103	,902
	Finland	Mean		14,3026	,70029
		95% Confidence Interval for Mean	Lower Bound	12,8849	
			Upper Bound	15,7202	
		5% Trimmed Mean		14,3028	
		Median		14,4000	
		Variance		19,126	
		Std. Deviation		4,37333	
		Minimum		7,20	
		Maximum		21,40	
		Range		14,20	
		Interquartile Range		7,90	
		Skewness		-,028	,378
		Kurtosis		-1,267	,741
	France	Mean		14,4436	,57101
		95% Confidence Interval for Mean	Lower Bound	13,2876	
			Upper Bound	15,5995	
		5% Trimmed Mean		14,3927	
		Median		14,1000	
		Variance		12,716	
		Std. Deviation		3,56598	
		Minimum		9,10	
		Maximum		20,80	
		Range		11,70	
		Interquartile Range		6,40	
		Skewness		,196	,378
		Kurtosis		-1,212	,741
	Germany	Mean		14,7769	,60261
		95% Confidence Interval for Mean	Lower Bound	13,5570	
			Upper Bound	15,9968	
		5% Trimmed Mean		14,7187	
		Median		14,5000	
		Variance		14,162	

Descriptives[a,b,c,d]

Country				Statistic	Std. Error
		Std. Deviation		3,76329	
		Minimum		9,20	
		Maximum		21,50	
		Range		12,30	
		Interquartile Range		6,70	
		Skewness		,187	,378
		Kurtosis		-1,218	,741
	Greece	Mean		18,0812	,61264
		95% Confidence Interval for Mean	Lower Bound	16,8318	
			Upper Bound	19,3307	
		5% Trimmed Mean		18,0625	
		Median		18,0000	
		Variance		12,011	
		Std. Deviation		3,46563	
		Minimum		12,50	
		Maximum		24,00	
		Range		11,50	
		Interquartile Range		6,10	
		Skewness		,079	,414
		Kurtosis		-1,184	,809
	Hungary	Mean		20,9217	,51633
		95% Confidence Interval for Mean	Lower Bound	19,8509	
			Upper Bound	21,9925	
		5% Trimmed Mean		20,8693	
		Median		20,5000	
		Variance		6,132	
		Std. Deviation		2,47624	
		Minimum		17,40	
		Maximum		25,40	
		Range		8,00	
		Interquartile Range		4,40	
		Skewness		,344	,481
		Kurtosis		-1,100	,935
	Ireland	Mean		14,9171	,85121
		95% Confidence Interval for Mean	Lower Bound	13,1873	
			Upper Bound	16,6470	
		5% Trimmed Mean		14,8579	
		Median		14,4000	

Descriptives[a,b,c,d]

Country				Statistic	Std. Error
		Variance		25,360	
		Std. Deviation		5,03584	
		Minimum		6,90	
		Maximum		24,00	
		Range		17,10	
		Interquartile Range		8,80	
		Skewness		,214	,398
		Kurtosis		-1,135	,778
	Italy	Mean		13,6949	,51799
		95% Confidence Interval for Mean	Lower Bound	12,6463	
			Upper Bound	14,7435	
		5% Trimmed Mean		13,6665	
		Median		13,5000	
		Variance		10,464	
		Std. Deviation		3,23484	
		Minimum		8,60	
		Maximum		19,30	
		Range		10,70	
		Interquartile Range		5,70	
		Skewness		,124	,378
		Kurtosis		-1,213	,741
	Latvia	Mean		21,0467	,30719
		95% Confidence Interval for Mean	Lower Bound	20,3878	
			Upper Bound	21,7055	
		5% Trimmed Mean		21,0352	
		Median		21,0000	
		Variance		1,416	
		Std. Deviation		1,18976	
		Minimum		19,30	
		Maximum		23,00	
		Range		3,70	
		Interquartile Range		2,10	
		Skewness		,133	,580
		Kurtosis		-1,185	1,121
	Luxembou	Mean		15,5063	,62757
		95% Confidence Interval for Mean	Lower Bound	14,2263	
			Upper Bound	16,7862	
		5% Trimmed Mean		15,4590	

Descriptives[a,b,c,d]

Country				Statistic	Std. Error
		Median		15,3000	
		Variance		12,603	
		Std. Deviation		3,55010	
		Minimum		10,20	
		Maximum		21,70	
		Range		11,50	
		Interquartile Range		6,33	
		Skewness		,182	,414
		Kurtosis		-1,228	,809
	Netherla	Mean		11,5769	,69090
		95% Confidence Interval for Mean	Lower Bound	10,1783	
			Upper Bound	12,9756	
		5% Trimmed Mean		11,4714	
		Median		10,9000	
		Variance		18,617	
		Std. Deviation		4,31469	
		Minimum		5,70	
		Maximum		19,50	
		Range		13,80	
		Interquartile Range		7,70	
		Skewness		,337	,378
		Kurtosis		-1,186	,741
	Norway	Mean		14,2897	,70409
		95% Confidence Interval for Mean	Lower Bound	12,8644	
			Upper Bound	15,7151	
		5% Trimmed Mean		14,2218	
		Median		13,8000	
		Variance		19,334	
		Std. Deviation		4,39706	
		Minimum		7,70	
		Maximum		22,20	
		Range		14,50	
		Interquartile Range		7,80	
		Skewness		,228	,378
		Kurtosis		-1,202	,741
	Poland	Mean		18,5957	,44373
		95% Confidence Interval for Mean	Lower Bound	17,6754	
			Upper Bound	19,5159	

Descriptives[a,b,c,d]

Country				Statistic	Std. Error
		5% Trimmed Mean		18,5681	
		Median		18,4000	
		Variance		4,529	
		Std. Deviation		2,12805	
		Minimum		15,40	
		Maximum		22,30	
		Range		6,90	
		Interquartile Range		3,80	
		Skewness		,187	,481
		Kurtosis		-1,191	,935
	Portugal	Mean		12,0103	,70714
		95% Confidence Interval for Mean	Lower Bound	10,5787	
			Upper Bound	13,4418	
		5% Trimmed Mean		11,9335	
		Median		11,6000	
		Variance		19,502	
		Std. Deviation		4,41611	
		Minimum		5,60	
		Maximum		19,90	
		Range		14,30	
		Interquartile Range		7,90	
		Skewness		,229	,378
		Kurtosis		-1,223	,741
	Slovak R	Mean		16,7000	,39352
		95% Confidence Interval for Mean	Lower Bound	15,8791	
			Upper Bound	17,5209	
		5% Trimmed Mean		16,6836	
		Median		16,6000	
		Variance		3,252	
		Std. Deviation		1,80333	
		Minimum		13,90	
		Maximum		19,80	
		Range		5,90	
		Interquartile Range		3,15	
		Skewness		,140	,501
		Kurtosis		-1,154	,972
	Slovenia	Mean		17,2000	,36121

Descriptives[a,b,c,d]

Country				Statistic	Std. Error
		95% Confidence Interval for Mean	Lower Bound	16,4253	
			Upper Bound	17,9747	
		5% Trimmed Mean		17,1889	
		Median		17,2000	
		Variance		1,957	
		Std. Deviation		1,39898	
		Minimum		15,10	
		Maximum		19,50	
		Range		4,40	
		Interquartile Range		2,50	
		Skewness		,113	,580
		Kurtosis		-1,200	1,121
	Spain	Mean		16,5974	,62170
		95% Confidence Interval for Mean	Lower Bound	15,3389	
			Upper Bound	17,8560	
		5% Trimmed Mean		16,5999	
		Median		16,6000	
		Variance		15,074	
		Std. Deviation		3,88252	
		Minimum		10,10	
		Maximum		23,10	
		Range		13,00	
		Interquartile Range		6,80	
		Skewness		-,010	,378
		Kurtosis		-1,190	,741
	Sweden	Mean		13,2821	,56555
		95% Confidence Interval for Mean	Lower Bound	12,1371	
			Upper Bound	14,4270	
		5% Trimmed Mean		13,2077	
		Median		12,9000	
		Variance		12,474	
		Std. Deviation		3,53188	
		Minimum		8,20	
		Maximum		19,80	
		Range		11,60	
		Interquartile Range		6,20	
		Skewness		,280	,378
		Kurtosis		-1,176	,741

Descriptives[a,b,c,d]

	Country			Statistic	Std. Error
	Switzerl	Mean		14,6792	,50408
		95% Confidence Interval for Mean	Lower Bound	13,6364	
			Upper Bound	15,7219	
		5% Trimmed Mean		14,6917	
		Median		14,6500	
		Variance		6,098	
		Std. Deviation		2,46946	
		Minimum		10,40	
		Maximum		18,70	
		Range		8,30	
		Interquartile Range		4,20	
		Skewness		-,039	,472
		Kurtosis		-1,076	,918
	United K	Mean		18,5290	,81370
		95% Confidence Interval for Mean	Lower Bound	16,8672	
			Upper Bound	20,1908	
		5% Trimmed Mean		18,4529	
		Median		18,1000	
		Variance		20,525	
		Std. Deviation		4,53050	
		Minimum		11,90	
		Maximum		26,60	
		Range		14,70	
		Interquartile Range		8,10	
		Skewness		,224	,421
		Kurtosis		-1,208	,821
unemplrate	Austria	Mean		4,5583	,16214
		95% Confidence Interval for Mean	Lower Bound	4,2201	
			Upper Bound	4,8965	
		5% Trimmed Mean		4,5514	
		Median		4,3114	
		Variance		,552	
		Std. Deviation		,74300	
		Minimum		3,53	
		Maximum		5,71	
		Range		2,18	
		Interquartile Range		1,46	
		Skewness		,151	,501

Descriptives[a,b,c,d]

Country				Statistic	Std. Error
		Kurtosis		-1,397	,972
	Belgium	Mean		8,6986	,26920
		95% Confidence Interval for Mean	Lower Bound	8,1496	
			Upper Bound	9,2477	
		5% Trimmed Mean		8,6350	
		Median		8,3951	
		Variance		2,319	
		Std. Deviation		1,52281	
		Minimum		6,64	
		Maximum		11,95	
		Range		5,31	
		Interquartile Range		1,97	
		Skewness		,784	,414
		Kurtosis		-,263	,809
	Czech Re	Mean		6,5115	,34308
		95% Confidence Interval for Mean	Lower Bound	5,7980	
			Upper Bound	7,2250	
		5% Trimmed Mean		6,5281	
		Median		6,9243	
		Variance		2,590	
		Std. Deviation		1,60921	
		Minimum		3,89	
		Maximum		8,83	
		Range		4,94	
		Interquartile Range		3,15	
		Skewness		-,357	,491
		Kurtosis		-1,180	,953
	Denmark	Mean		6,6725	,33323
		95% Confidence Interval for Mean	Lower Bound	5,9928	
			Upper Bound	7,3521	
		5% Trimmed Mean		6,6291	
		Median		6,6743	
		Variance		3,553	
		Std. Deviation		1,88504	
		Minimum		3,48	
		Maximum		10,87	
		Range		7,39	
		Interquartile Range		2,97	

Descriptives[a,b,c,d]

Country				Statistic	Std. Error
		Skewness		,263	,414
		Kurtosis		-,641	,809
	Estonia	Mean		9,0709	,79649
		95% Confidence Interval for Mean	Lower Bound	7,4270	
			Upper Bound	10,7148	
		5% Trimmed Mean		9,1111	
		Median		9,7496	
		Variance		15,860	
		Std. Deviation		3,98246	
		Minimum		,63	
		Maximum		17,07	
		Range		16,44	
		Interquartile Range		5,56	
		Skewness		-,279	,464
		Kurtosis		,005	,902
	Finland	Mean		8,0999	,55094
		95% Confidence Interval for Mean	Lower Bound	6,9845	
			Upper Bound	9,2152	
		5% Trimmed Mean		7,9128	
		Median		7,7560	
		Variance		11,838	
		Std. Deviation		3,44062	
		Minimum		3,09	
		Maximum		16,53	
		Range		13,43	
		Interquartile Range		3,78	
		Skewness		,955	,378
		Kurtosis		,492	,741
	France	Mean		9,0762	,32376
		95% Confidence Interval for Mean	Lower Bound	8,4208	
			Upper Bound	9,7317	
		5% Trimmed Mean		9,1345	
		Median		9,0822	
		Variance		4,088	
		Std. Deviation		2,02190	
		Minimum		4,57	
		Maximum		12,40	
		Range		7,83	

Descriptives[a,b,c,d]

Country				Statistic	Std. Error
		Interquartile Range		2,14	
		Skewness		-,452	,378
		Kurtosis		-,070	,741
	Germany	Mean		7,0076	,33305
		95% Confidence Interval for Mean	Lower Bound	6,3334	
			Upper Bound	7,6819	
		5% Trimmed Mean		7,0033	
		Median		7,1608	
		Variance		4,326	
		Std. Deviation		2,07993	
		Minimum		3,23	
		Maximum		11,28	
		Range		8,05	
		Interquartile Range		3,01	
		Skewness		-,046	,378
		Kurtosis		-,675	,741
	Greece	Mean		11,1924	,95410
		95% Confidence Interval for Mean	Lower Bound	9,2465	
			Upper Bound	13,1383	
		5% Trimmed Mean		10,5101	
		Median		9,5477	
		Variance		29,130	
		Std. Deviation		5,39720	
		Minimum		7,20	
		Maximum		27,70	
		Range		20,49	
		Interquartile Range		2,95	
		Skewness		2,306	,414
		Kurtosis		4,492	,809
	Hungary	Mean		8,6159	,42308
		95% Confidence Interval for Mean	Lower Bound	7,7385	
			Upper Bound	9,4933	
		5% Trimmed Mean		8,5893	
		Median		7,8664	
		Variance		4,117	
		Std. Deviation		2,02903	
		Minimum		5,73	
		Maximum		12,08	

Descriptives[a,b,c,d]

Country				Statistic	Std. Error
		Range		6,35	
		Interquartile Range		3,24	
		Skewness		,087	,481
		Kurtosis		-1,422	,935
	Ireland	Mean		11,4117	,79406
		95% Confidence Interval for Mean	Lower Bound	9,7980	
			Upper Bound	13,0254	
		5% Trimmed Mean		11,4565	
		Median		12,7120	
		Variance		22,068	
		Std. Deviation		4,69769	
		Minimum		4,19	
		Maximum		17,70	
		Range		13,51	
		Interquartile Range		9,36	
		Skewness		-,325	,398
		Kurtosis		-1,537	,778
	Italy	Mean		9,6355	,34599
		95% Confidence Interval for Mean	Lower Bound	8,9351	
			Upper Bound	10,3359	
		5% Trimmed Mean		9,7310	
		Median		10,0636	
		Variance		4,669	
		Std. Deviation		2,16070	
		Minimum		3,75	
		Maximum		12,90	
		Range		9,14	
		Interquartile Range		3,84	
		Skewness		-,516	,378
		Kurtosis		-,348	,741
	Latvia	Mean		12,6124	,99876
		95% Confidence Interval for Mean	Lower Bound	10,4703	
			Upper Bound	14,7545	
		5% Trimmed Mean		12,5694	
		Median		12,1071	
		Variance		14,963	
		Std. Deviation		3,86816	
		Minimum		6,19	

Descriptives[a,b,c,d]

Country				Statistic	Std. Error
		Maximum		19,81	
		Range		13,62	
		Interquartile Range		5,19	
		Skewness		,108	,580
		Kurtosis		-,411	1,121
	Luxembou	Mean		3,3705	,23675
		95% Confidence Interval for Mean	Lower Bound	2,8877	
			Upper Bound	3,8534	
		5% Trimmed Mean		3,3335	
		Median		2,9714	
		Variance		1,794	
		Std. Deviation		1,33924	
		Minimum		1,49	
		Maximum		5,91	
		Range		4,42	
		Interquartile Range		2,32	
		Skewness		,433	,414
		Kurtosis		-1,065	,809
	Netherla	Mean		6,4329	,47629
		95% Confidence Interval for Mean	Lower Bound	5,4687	
			Upper Bound	7,3971	
		5% Trimmed Mean		6,2053	
		Median		5,6781	
		Variance		8,847	
		Std. Deviation		2,97442	
		Minimum		2,51	
		Maximum		14,32	
		Range		11,81	
		Interquartile Range		3,19	
		Skewness		1,296	,378
		Kurtosis		1,240	,741
	Norway	Mean		3,5381	,19900
		95% Confidence Interval for Mean	Lower Bound	3,1353	
			Upper Bound	3,9410	
		5% Trimmed Mean		3,5068	
		Median		3,4557	
		Variance		1,544	
		Std. Deviation		1,24277	

Descriptives[a,b,c,d]

Country				Statistic	Std. Error
		Minimum		1,54	
		Maximum		6,06	
		Range		4,53	
		Interquartile Range		1,90	
		Skewness		,334	,378
		Kurtosis		-,667	,741
	Poland	Mean		13,2497	,82308
		95% Confidence Interval for Mean	Lower Bound	11,5428	
			Upper Bound	14,9567	
		5% Trimmed Mean		13,1896	
		Median		12,7897	
		Variance		15,581	
		Std. Deviation		3,94734	
		Minimum		7,20	
		Maximum		20,28	
		Range		13,08	
		Interquartile Range		6,61	
		Skewness		,438	,481
		Kurtosis		-,942	,935
	Portugal	Mean		7,7067	,49516
		95% Confidence Interval for Mean	Lower Bound	6,7043	
			Upper Bound	8,7091	
		5% Trimmed Mean		7,4045	
		Median		7,0926	
		Variance		9,562	
		Std. Deviation		3,09229	
		Minimum		4,15	
		Maximum		17,00	
		Range		12,85	
		Interquartile Range		2,78	
		Skewness		1,599	,378
		Kurtosis		2,575	,741
	Slovak R	Mean		14,4441	,61048
		95% Confidence Interval for Mean	Lower Bound	13,1707	
			Upper Bound	15,7175	
		5% Trimmed Mean		14,4395	
		Median		13,6707	
		Variance		7,826	

Descriptives[a,b,c,d]

Country				Statistic	Std. Error
		Std. Deviation		2,79758	
		Minimum		9,60	
		Maximum		19,33	
		Range		9,73	
		Interquartile Range		4,60	
		Skewness		,357	,501
		Kurtosis		-,847	,972
	Slovenia	Mean		7,0677	,42971
		95% Confidence Interval for Mean	Lower Bound	6,1461	
			Upper Bound	7,9893	
		5% Trimmed Mean		7,0339	
		Median		6,6654	
		Variance		2,770	
		Std. Deviation		1,66426	
		Minimum		4,46	
		Maximum		10,28	
		Range		5,82	
		Interquartile Range		2,24	
		Skewness		,627	,580
		Kurtosis		-,085	1,121
	Spain	Mean		16,1575	,94345
		95% Confidence Interval for Mean	Lower Bound	14,2476	
			Upper Bound	18,0674	
		5% Trimmed Mean		16,2584	
		Median		17,1179	
		Variance		34,714	
		Std. Deviation		5,89182	
		Minimum		4,62	
		Maximum		26,22	
		Range		21,60	
		Interquartile Range		9,52	
		Skewness		-,248	,378
		Kurtosis		-,975	,741
	Sweden	Mean		5,5707	,44988
		95% Confidence Interval for Mean	Lower Bound	4,6599	
			Upper Bound	6,4814	
		5% Trimmed Mean		5,5373	
		Median		5,8368	

Descriptives[a,b,c,d]

Country				Statistic	Std. Error
		Variance		7,893	
		Std. Deviation		2,80953	
		Minimum		1,62	
		Maximum		10,15	
		Range		8,53	
		Interquartile Range		5,33	
		Skewness		,049	,378
		Kurtosis		-1,457	,741
	Switzerl	Mean		3,8017	,16796
		95% Confidence Interval for Mean	Lower Bound	3,4542	
			Upper Bound	4,1491	
		5% Trimmed Mean		3,8405	
		Median		3,8977	
		Variance		,677	
		Std. Deviation		,82286	
		Minimum		1,82	
		Maximum		4,97	
		Range		3,15	
		Interquartile Range		1,34	
		Skewness		-,596	,472
		Kurtosis		-,114	,918
	United K	Mean		7,5615	,38929
		95% Confidence Interval for Mean	Lower Bound	6,7665	
			Upper Bound	8,3565	
		5% Trimmed Mean		7,4917	
		Median		7,6823	
		Variance		4,698	
		Std. Deviation		2,16750	
		Minimum		4,70	
		Maximum		11,86	
		Range		7,16	
		Interquartile Range		3,41	
		Skewness		,408	,421
		Kurtosis		-,870	,821

a. There are no valid cases for Fertility when Country = 6,789E+199. Statistics cannot be computed for this level.

b. There are no valid cases for Overweight when Country = 6,789E+199. Statistics cannot be computed for this level.

c. There are no valid cases for Obesity when Country = 6,789E+199. Statistics cannot be computed for this level.

d. There are no valid cases for unemplrate when Country = 6,789E+199. Statistics cannot be computed for this level.

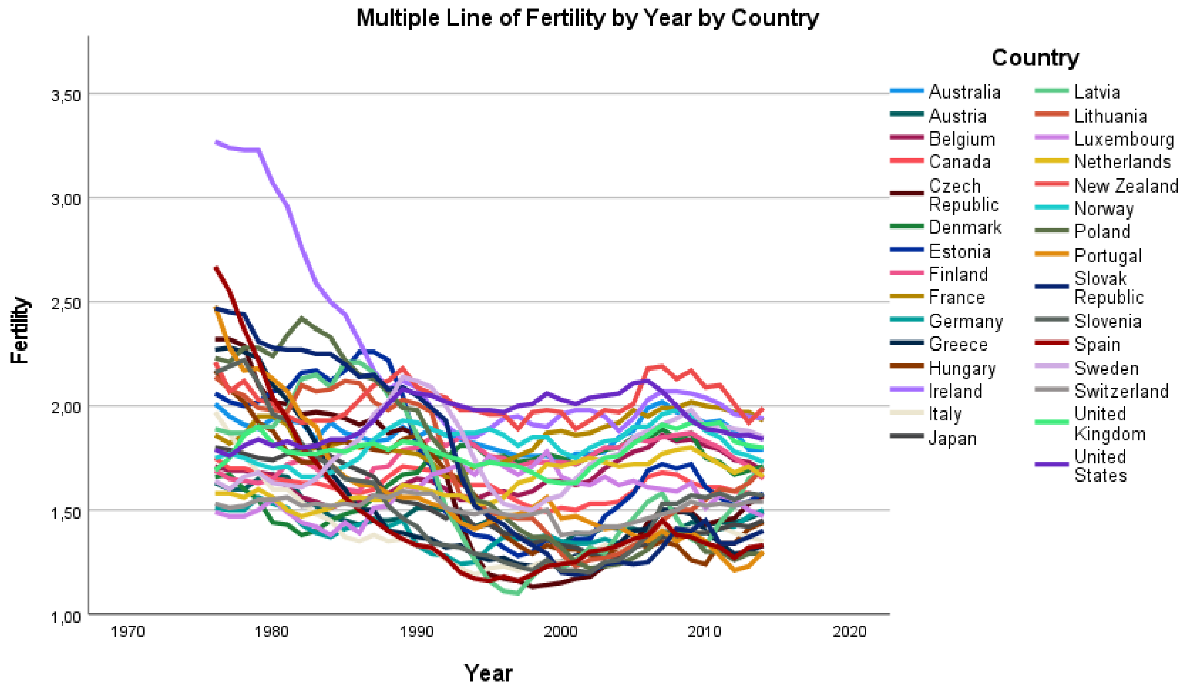

Figure S1. Fertility rate (children per women) per country per year from 1976 until 2014.

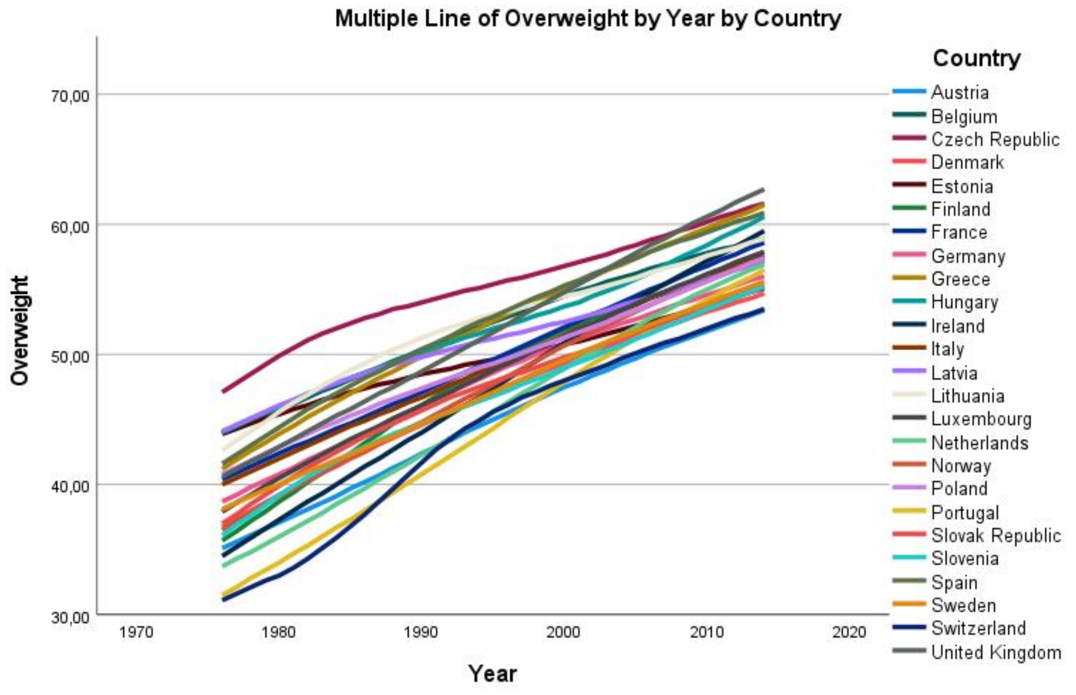

Figure S2. Overweight rate per country per year from 1976 until 2014.

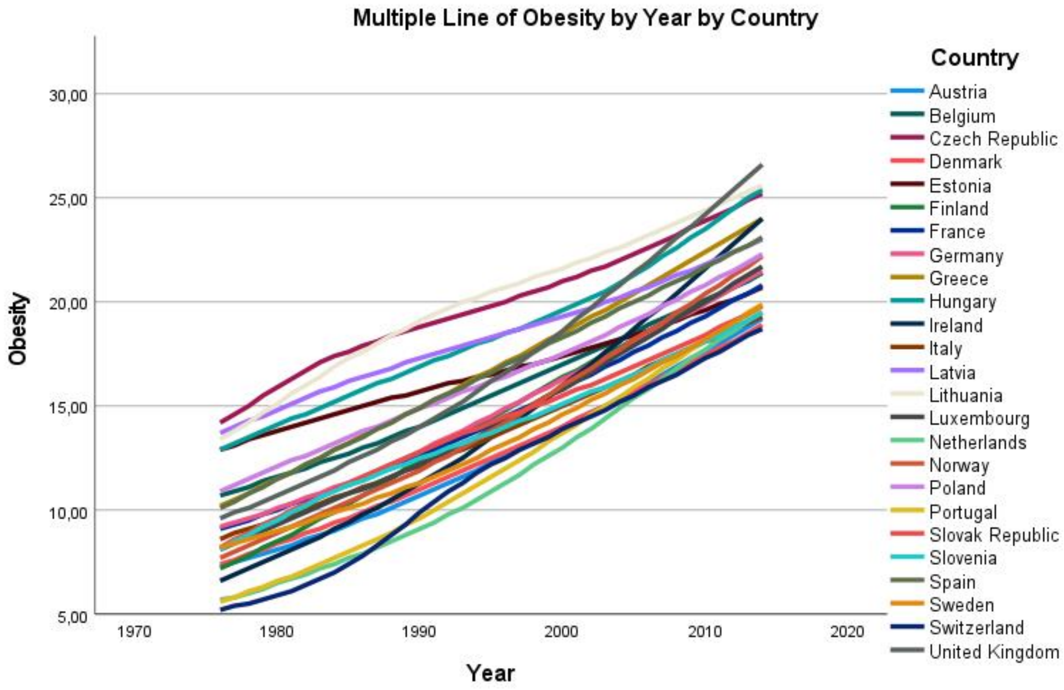

Figure S3. Obesity rate per country per year from 1976 until 2014.

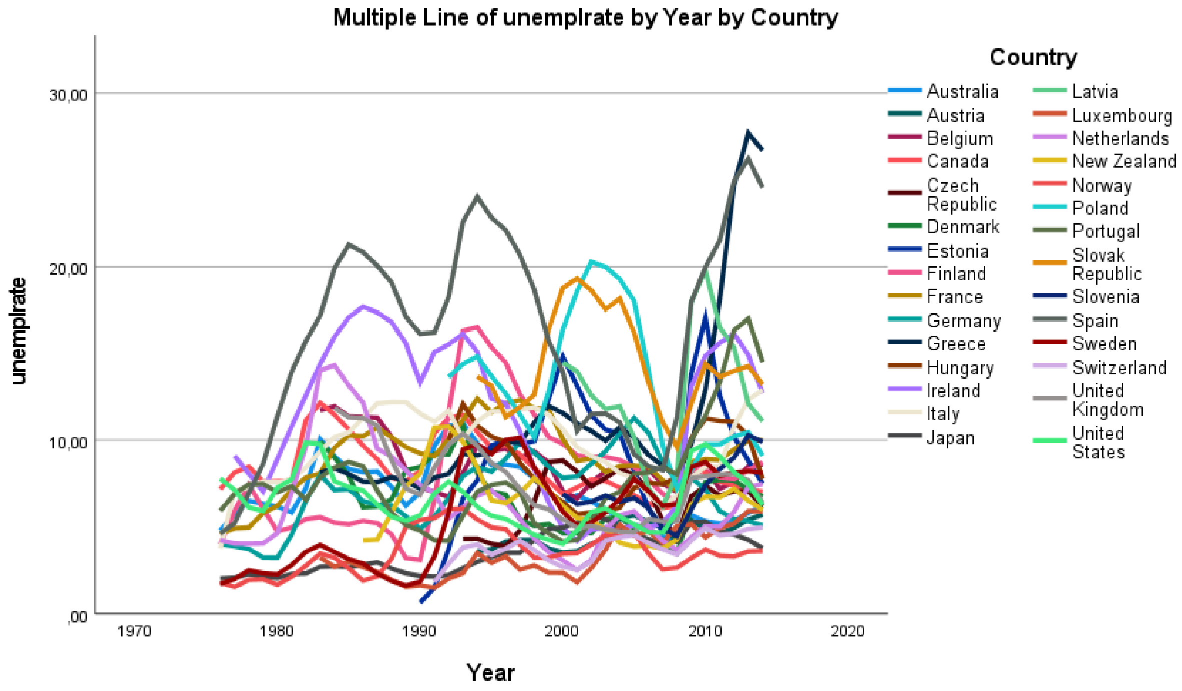

Figure S4. Unemployment rate (number of individuals of the population of working age registered as being unemployed) per country per year from 1976 until 2014.
